# Low visual information-processing speed and attention are predictors of fatigue in elementary and junior high school students

**DOI:** 10.1186/1744-9081-7-20

**Published:** 2011-06-14

**Authors:** Kei Mizuno, Masaaki Tanaka, Sanae Fukuda, Emi Yamano, Yoshihito Shigihara, Kyoko Imai-Matsumura, Yasuyoshi Watanabe

**Affiliations:** 1 Department of Physiology, Osaka City University Graduate School of Medicine, 1-4-3 Asahimachi, Abeno-ku, Osaka City, Osaka 545-8585, Japan; 2 Molecular Probe Dynamics Laboratory, RIKEN Center for Molecular Imaging Science, 6-7-3 Minatojima-minamimachi, Chuo-ku, Kobe City, Hyogo 650-0047, Japan; 3 Department of Medical Science on Fatigue, Osaka City University Graduate School of Medicine, 1-4-3 Asahimachi, Abeno-ku, Osaka City, Osaka 545-8585, Japan; 4 Department of Clinical, Health and Special Needs Education Needs, Hyogo University of Teacher Education, Graduate School of Education, 942-1 Shimokume, Kato City, Hyogo 673-1494, Japan

## Abstract

**Background:**

Fatigue is a common complaint among elementary and junior high school students, and is known to be associated with reduced academic performance. Recently, we demonstrated that fatigue was correlated with decreased cognitive function in these students. However, no studies have identified cognitive predictors of fatigue. Therefore, we attempted to determine independent cognitive predictors of fatigue in these students.

**Methods:**

We performed a prospective cohort study. One hundred and forty-two elementary and junior high school students without fatigue participated. They completed a variety of paper-and-pencil tests, including list learning and list recall tests, *kana *pick-out test, semantic fluency test, figure copying test, digit span forward test, and symbol digit modalities test. The participants also completed computerized cognitive tests (tasks A to E on the modified advanced trail making test). These cognitive tests were used to evaluate motor- and information-processing speed, immediate and delayed memory function, auditory and visual attention, divided and switching attention, retrieval of learned material, and spatial construction. One year after the tests, a questionnaire about fatigue (Japanese version of the Chalder Fatigue Scale) was administered to all the participants.

**Results:**

After the follow-up period, we confirmed 40 cases of fatigue among 118 students. In multivariate logistic regression analyses adjusted for grades and gender, poorer performance on visual information-processing speed and attention tasks was associated with increased risk of fatigue.

**Conclusions:**

Reduced visual information-processing speed and poor attention are independent predictors of fatigue in elementary and junior high school students.

## Background

Fatigue refers to the feeling that people may experience after or during prolonged activity [1]. Fatigue is a common symptom among students. Up to 8% of children and adolescents have experienced fatigue for more than one month, and nearly 2% have experienced chronic fatigue lasting more than six months [2]. Because fatigue in students is associated with a decrease in academic performance [3], the impact of fatigue on children and adolescents requires additional attention.

When students proceed to junior high school from elementary school, a multitude of changes occur in their environment, which have the potential to cause a variety of behavioral and emotional problems [4]. One example is the number of Japanese students exhibiting school refusal, which was observed in 0.6% of 6th-graders and 1.9% of 7th-graders in 2005 [5]. It has been reported that student fatigue also markedly increases from elementary school to junior high school [6]. Identifying fatigue-related factors is thus important for preventing increased levels of fatigue during this transition period.

Executive function is defined as a set of cognitive control processes that permit goal-directed behavior and that develop dramatically between childhood and adolescence [7]. In studies on the development of executive function, for example, age-related gain has been reported in working memory [8,9], inhibitory control [10], task switching [11], control of attention [12], adaptive problem solving [13], and various other planning and problem-solving tasks [14]. Recently, we demonstrated the development of several cognitive functions in elementary school and junior high school students using paper-and-pencil and computerized cognitive function tests which are used in the present study as well [15]. Task performance of students on an auditory attention task (digit span forward test), visual information-processing speed and attention tasks [symbol digit modalities test and tasks A and B on the modified advanced trail making test (mATMT)], retrieval of learned material task (semantic fluency test), switching attention task (task E on the mATMT), and a divided attention task (*kana *pick-out test) improved from elementary to junior high school. Thus, these cognitive functions develop from childhood to adolescence. Based on these findings, these cognitive tests were advantageous in the present study for evaluation of cognitive development in children and adolescents.

Fatigue has been shown to be associated with impaired cognitive function in studies of adults [16-19]. In children and adolescents, childhood chronic fatigue syndrome (CCFS), characterized by profound and disabling fatigue persisting for at least 6 months [20], can severely impair cognitive functions such as learning, short-term memory, visual information-processing speed and attention processing [21-23]. From these data, impaired cognitive function appeared to be associated with fatigue; however, these findings were limited to a specific disease. Therefore, we recently demonstrated that fatigue was also correlated with reduced cognitive function in elementary and junior high school students. Slow visual motor processing was positively correlated with the prevalence of fatigue in the elementary school students and decreased visual information-processing speed and attention processing were positively correlated with the prevalence of fatigue in junior high school students [24]. Although a relationship between fatigue and cognitive function has been established, no studies to date have identified cognitive predictors of fatigue in these students. The ability to identify these predictors might help education professionals to develop screening procedures to identify those at high risk for fatigue, and to conduct early interventions to achieve lower incidences of and higher rates of recovery from fatigue. Therefore, in the present prospective cohort study, we examined whether some low cognitive functions were independently associated with the risk of fatigue in elementary and junior high school students.

## Methods

### Participants

Participants were enrolled from the 4th, 5th, and 6th grades in an elementary school and from 7th, 8th, and 9th grades in a junior high school in Hyogo Prefecture, Japan, between November 2006 and December 2006 [15]. Most of the students at the elementary school were expected to proceed on to the junior high school. Among 362 participants, 45 students with medical illnesses, such as allergic disease, bronchial asthma, thyroid disease, nephritis, diabetes mellitus, heart disease, anemia, myodystrophy, and epilepsy, were excluded from the analyses. In addition, 17 students who did not complete the fatigue questionnaire were also excluded. Furthermore, 103 fatigued participants and 55 9th grade junior high school students, who were to be graduated from the school after 1 year, were also excluded. Among the 142 participants, 118 cases (83.1%) were entered in a 1-year prospective fatigue registry, between November 2007 and December 2007, and 24 did not meet eligibility requirement because of the failure to complete the fatigue questionnaire. The study protocol was approved by the Ethics Committee of Osaka City University, and all the participants and their parents gave written informed consent to participate in the study.

### Fatigue scale

The severity of fatigue was measured using the Chalder Fatigue Scale [25]. The Chalder Fatigue Scale questionnaire was distributed to the students in a classroom at their school before the cognitive tests. The reliability and validity of the Japanese version of the Chalder Fatigue Scale for evaluation of the severity of fatigue in students were previously confirmed [26]. The fatigue scale consists of 11 questions using a 2-level (0-1) general health questionnaire scale in which responses can be 0 (less than usual or no more than usual) or 1 (more than usual or much more than usual) during the past several weeks. The total score for the 11-item fatigue scale ranges from 0 to 11, with higher scores indicating a greater degree of fatigue. Fatigue was defined as a score of equal to or more than 4 on the Chalder Fatigue Scale [25].

### Cognitive function tests

Students performed a variety of paper-and-pencil and computerized cognitive tests [15,27]. Participants were given an explanation of the rules for each cognitive test, and before the participants performed each cognitive test, they practiced for 1 min. Half of the participants performed the paper-and-pencil cognitive tests in the classroom for around 35 min. After the paper-and-pencil cognitive tests, participants moved to a computer room and performed the computerized cognitive tests within 10 min. The other half of the participants first performed the computerized cognitive tests in the computer room, and then moved to the classroom and performed the paper-and-pencil cognitive tests.

The paper-and-pencil cognitive tests consisted of a list learning test [28], *kana *pick-out test [23], semantic fluency test [29], figure copying test [28], digit span forward test [30], symbol digit modalities test [31], and list recall test [28], performed in this order.

The list learning test was used to assess immediate memory. This test consists of immediate recall of a 10-item list of words over four learning trials. The words are semantically unrelated, characterized by early age of acquisition and relatively high-level imagery, and were as phonetically unique as possible. The full score for this test is 40.

The *kana *pick-out test requires parallel processing of reading and picking out of letters, and also requires appropriate allocation of attentional resources to the two activities. Participants are shown a short story written in Japanese *kana *characters. They are required to find as many vowel symbols as possible within 2 min, while understanding the meaning of the story. Two min after the start of the test, they are asked 10 questions about the contents of the story over a 2 min period. The Japanese *kana *characters consist of 66 phonetic symbols that include five vowels; the story consists of 406 symbols with 61 vowels. The *kana *pick-out score is calculated as the number of *kana *characters picked out minus the number of *kana *characters missed. The full *kana *pick-out score for this test is 61, with a full comprehension score of 10.

The semantic fluency test was used to assess retrieval of learned material. This test consists of the total number of exemplars generated for a given semantic category (vegetables) within 1 min. The semantic categories were chosen in an attempt to minimize retrieval demands and thereby more specifically tap semantic stores rather than retrieval strategies.

The figure copying test was used to assess spatial construction ability. This test consists of copying of a geometric figure comprised of 10 parts. Each part receives a 2-point score (accuracy and placement), for a total of 20 possible points, and thus a possible full score of 20. The time limit for performance of the test is set at 4 min.

The digit span forward test was used primarily to assess auditory attention. An experimenter read a series of digits at the rate of approximately one number per second and asked the participants to write on paper the digits exactly as read. The series ranged from 4 to 8 digits in length and were presented in order of increasing numbers of digits. The full score for this test is 8.

The cognitive demand of the symbol digit modalities test primarily includes visual information-processing speed and attention. The test consists of 1) a key with two rows, with nine stimulus symbols in the upper row, and matched numbers (1 - 9) in the lower row, and 2) a two-row grid with the same nine stimulus symbols in the upper row and 84 blank cells for numeric responses in the lower row. The full score for this test is 84. The time limit for performance of this test is set at 1 min and 30 s.

The list recall test was used to assess delayed memory. This test involves free recall of the words from the list learning test. The list recall test was performed 30 min after the list learning test. The full score for this test is 10.

The computerized cognitive test consisted of the mATMT and separate tasks A, B, C, D, and E [32,33]. For the mATMT, participants performed visual search trials. In this test, circles numbered from 1 to 25 are randomly located on the screen of a personal computer, and participants are required to use a computer mouse to click on these circles in sequence, starting with circle number 1. In task A, when the participant clicks on a target circle, the positions of the other circles also remain the same. In task B, when the participant clicks on the first target circle, a new circle number 26 appears on the screen. The positions of the other circles remain the same. Thus, tasks A and B primarily require visual information-processing speed and attention. The procedure for task C is the same as that for task B, except that the positions of the other circles randomly change after each click on a target circle. Thus, task C primarily requires visual search rather than visual information-processing speed and attention. In task D, circles numbered from 1 to 25 are regularly and sequentially located on a computer screen. Thus, task D requires no visual search, and only visual motor processing. In task E, circles numbered from 1 to 13 and 12 *kana *letters (Japanese phonograms) are randomly located on the screen, and participants are required to use a computer mouse to alternately click on numbers and *kana*; this task thus requires switching attention. Participants performed tasks A, B, C, D, and E in this order, and time limits for performance of the mATMT were set at 10 min. Participants were instructed to perform the tasks as quickly and accurately as possible. In order to assess visual information-processing speed and attention, visual search, and attention switching, excluding effects of visual motor processing, we calculated differences in reaction time as total reaction times on task A, B, C, or E subtracted by that on task D in the mATMT [15].

### Statistical analyses

The presented values are shown as the mean ± standard deviation (SD) unless otherwise stated. We used univariate and multivariate logistic regression analyses to estimate the odds ratio (OR) per 1 SD increase of each cognitive test score for the incidence of fatigue in relation to baseline cognitive variables in the elementary and junior high school students. We calculated the 95% confidence interval (CI) for each OR and all *p *values were two-tailed. A *p *value less than .05 was considered statistically significant. Statistical analyses were performed using the SPSS 17.0 software package (SPSS, Chicago, IL).

## Results

Baseline characteristics of the study students are summarized in Tables 1 and 2. The 118 elementary and junior high school students were not fatigued (fatigue score, 1.5 ± 1.2), because fatigued students who had the score of equal to or more than 4 on the Chalder Fatigue Scale [25], had been excluded from the analysis. Among the eligible 118 elementary and junior high school students followed for 1 year, 40 (33.9%) developed fatigue (fatigues score, 5.9 ± 1.4) and 78 (66.1%) did not develop fatigue (fatigue score, 1.1 ± 1.1).

**Table 1:** Baseline characteristics of the five grades analyzed

	Elementary school			Junior high school	
	
Grade level	4th grade	5th grade	6th grade	7th grade	8th grade
Age range	(9 - 10 years)	(10 - 11 years)	(11 - 12 years)	(12 - 13 years)	(13 - 14 years)
Age (years)	9.7 ± 0.5	10.7 ± 0.5	11.7 ± 0.5	12.6 ± 0.5	13.4 ± 0.5
Female/Male	16/16	15/10	16/10	16/9	0/10

Values are presented as the mean ± SD or number.

**Table 2:** Baseline characteristics of study participants (n = 118)

Age (years)	11.3 ± 1.3
Female (%)	63 (53.4)
Chalder fatigue scale (score)	1.5 ± 1.2
List learning test (score)	31.9 ± 3.6
List recall test (score)	8.6 ± 1.4
Digit span forward test (score)	5.9 ± 1.1
Symbol digit modalities test (score)	49.2 ± 13.2
Semantic fluency test (score)	9.8 ± 2.6
Figure copying test (score)	17.3 ± 2.8
*Kana *pick-out test	
*Kana *pick-out number	30.9 ± 9.8
*Kana *omission number	7.6 ± 6.9
*Kana *pick-out score	23.3 ± 11.8
Score for story comprehension	4.0 ± 2.0
Modified advanced trail making test	
RT on task A (s)	42.6 ± 13.3
RT on task B (s)	62.6 ± 18.0
RT on task C (s)	87.1 ± 24.3
RT on task D (s)	14.2 ± 4.2
RT on task E (s)	72.9 ± 32.4
RT on task A-D (s)	28.4 ± 12.0
RT on task B-D (s)	48.4 ± 16.4
RT on task C-D (s)	72.9 ± 22.7
RT on task E-D (s)	58.7 ± 30.4

RT, reaction time. Values are presented as the mean ± SD or number (%).

In order to identify cognitive predictors associated with fatigue in the elementary and junior high school students, univariate and multivariate logistic regression analyses were performed (Table 3). Although, in univariate logistic regression analyses, no cognitive tests reached statistical significance, in multivariate logistic regression analyses adjusted for grades and gender, lower scores on the symbol digit modalities test [OR: 1.85, 95% CI: 3.03 to 1.12 (per 1 SD decrease), *p *= .016] and longer reaction time on task A of mATMT [OR: 1.88, 95% CI: 1.20 to 2.94 (per 1 SD increase), *p *= .006] were associated with a higher risk of fatigue in the elementary and junior high school students. In addition, longer reaction times on task B showed a trend toward increasing risk of fatigue in these students [OR: 1.66, 95% CI: 0.97 to 2.85 (per 1 SD increase), *p *= .065]. No other scores of cognitive tests were predictors of fatigue in these students.

**Table 3:** Univariate and multivariate logistic regression analyses of the occurrence of fatigue in elementary and junior high school students

	OR (95% CI)	
	
	Crude	Multiple-Adjusted*
Grade		
4th grade	Reference (1.00)	
5th grade	1.22 (0.37 - 3.96)	
6th grade	1.39 (0.44 - 4.40)	
7th grade	2.08 (0.67 - 6.44)	
8th grade	7.29 (1.52 - 35.03)	
*p *for trend	0.005	
Male	1.19 (0.56 - 2.56)	
List learning test	0.93 (0.63 - 1.37)	0.82 (0.54 - 1.22)
List recall test	0.91 (0.64 - 1.29)	0.79 (0.54 - 1.15)
Digit span forward test	1.06 (0.72 - 1.55)	0.87 (0.56 - 1.36)
Symbol digit modalities test	0.81 (0.55 - 1.20)	0.54 (0.33 - 0.89)
Semantic fluency test	0.87 (0.58 - 1.37)	0.70 (0.43 - 1.13)
Figure copying test	0.89 (0.62 - 1.27)	0.77 (0.53 - 1.13)
*Kana *pick-out test		
*Kana *pick-out number	1.00 (0.67 - 1.50)	0.64 (0.37 - 1.10)
*Kana *omission number	0.91 (0.61 - 1.37)	0.87 (0.56 - 1.35)
*Kana *pick-out score	1.06 (0.70 - 1.61)	0.81 (0.50 - 1.32)
Score for story comprehension	0.89 (0.61 - 1.30)	0.87 (0.57 - 1.32)
Modified advanced trail making test		
RT on task A (per 1 SD increase)	1.38 (0.97 - 1.98)	1.88 (1.20 - 2.94)
RT on task B (per 1 SD increase)	1.21 (0.79 - 1.85)	1.66 (0.97 - 2.85)
RT on task C (per 1 SD increase)	0.99 (0.65 - 1.51)	1.21 (0.75 - 1.95)
RT on task E (per 1 SD increase)	1.03 (0.75 - 1.43)	1.26 (0.88 - 1.81)
RT on task A-D (per 1 SD increase)	1.29 (0.91 - 1.81)	1.62 (1.08 - 2.43)
RT on task B-D (per 1 SD increase)	1.13 (0.74 - 1.72)	1.44 (0.86 - 2.42)
RT on task C-D (per 1 SD increase)	0.72 (0.60 - 1.43)	1.10 (0.68 - 1.77)
RT on task E-D (per 1 SD increase)	1.00 (0.72 - 1.38)	1.20 (0.84 - 1.72)

*Adjusted for grade and gender. RT, reaction time; OR, odds ratio; CI, confidence interval.

In task A of mATMT, even after the subtraction of the reaction time associated with motor processing (reaction time on task D), multivariate logistic regression analyses showed that longer reaction time was a predictor of fatigue in the elementary and junior high school students [OR: 1.62, 95% CI: 1.08 to 2.43 (per 1 SD increase); *p *= .019; Table 3]. No other scores of cognitive tests were predictors of fatigue in these students.

## Discussion

These prospective data demonstrate that lower scores on the symbol digit modalities test and longer reaction times on tasks A and B of the mATMT were associated with the risk of fatigue in elementary and junior high school students. These findings were independent of grade and gender. To our knowledge, this prospective cohort study provides the first evidence identifying cognitive risk factors of fatigue in students.

Tasks A and B of the mATMT primarily require visual information-processing speed and attention. In contrast, task C requires visual search rather than visual information-processing speed and attention. Although longer reaction times on tasks A and B were associated with increased risk of fatigue, longer reaction times on task C were not associated with increased risk of fatigue in the elementary and junior high school students. In addition, a lower score on the symbol digit modalities test, which was also used to assess visual information-processing speed and attention, was associated with increased risk of fatigue in these students. These results suggest that low visual information-processing speed and attention are predictors of fatigue in children and adolescents.

Fatigue is defined as the difficulty in initiating or sustaining voluntary activities [34], suggesting that abilities or capacities for initiating and sustaining cognitive processing are important to maintain an unwearied condition. In the present study, the multiple logistic regression analyses revealed that poor performance on visual information-processing speed and attention tasks were predictors of fatigue in elementary and junior high school students. These results suggest that well-developed abilities of visual information-processing speed and attention are beneficial to prevent the development of fatigue in these students. Since these cognitive performances improve from elementary to junior high school [15], our findings in the present study might help education professionals to develop screening procedures to identify not only cognitive developmental milestones but also cognitive functions at high risk for fatigue, and to conduct early interventions to achieve lower incidences of and higher rates of recovery from fatigue.

We did not identify a mechanism by which low visual information-processing speed and attention increased the risk of fatigue. In patients with CFS [26], the relationship between fatigue and deficits of visual information-processing speed and attention in accordance with symbol digit modalities test [35-37] and task A on TMT [37,38] have been shown. In a functional magnetic resonance imaging study, the lateral prefrontal and parietal cortices were activated during both tasks A and B on TMT [39]. Patients with CFS showed greater activation in the frontal cortex than healthy participants during a relatively easy task. In contrast, during a more challenging task, patients with CFS demonstrated reduced activations in the lateral prefrontal and parietal cortices [40]. In addition, there is reduced gray-matter volume in the lateral prefrontal cortex in patients with CFS [41,42], indicating that the neural pathophysiology of CFS may be associated with more demanding cognitive processing [40]. The development of cognitive functions is observed in association with structural changes in the brain. Morphological analyses in children and adolescents have shown that brain maturation occurs at different rates in different brain regions: the primary sensory and motor areas are the first to complete development, while the association areas, especially in the frontal and parietal regions, are the last to mature [43]. Maturation of the frontal and parietal regions is of great importance for adequate processing of developed cognitive functions which require activation of these brain regions. In fact, task performance on visual information-processing speed and attention improves from elementary to junior high school [15]. Although findings of patients with CFS by neuroimaging and morphometry studies were limited to a specific adult disease, and further studies are necessary to determine whether these findings can be generalized to healthy elementary and junior high school students, impaired development of frontal and parietal brain regions might introduce fatigue in the elementary and junior high school students.

## Limitations

The present study has two limitations. First, we performed this study with a limited number of participants. To generalize our results, studies involving a larger number of participants are essential. Second, we did not exclude developmental disabilities from analyses and did not assess the socioeconomic status and intelligence quotient.

## Conclusions

The present results provide evidence that low visual information-processing speed and attention are independent predictors of fatigue in elementary and junior high school students. Fatigue is associated with impairment of academic performance [3] in elementary and junior high school students. Therefore, in order to prevent the development of fatigue leading to impaired academic performance in these students, early identification of the students at high risk for fatigue is important. To this end, the symbol digit modalities test and mATMT are appropriate for the evaluation of cognitive functions associated with assessment of the risk of fatigue in elementary and junior high school students.

## List of abbreviations

CCFS: Childhood chronic fatigue syndrome; CI: Confidence interval; mATMT: Modified advanced trail making test; OR: Odds ratio; RT: Reaction time; SD: Standard deviation.

## Author details

^1^Department of Physiology, Osaka City University Graduate School of Medicine, 1-4-3 Asahimachi, Abeno-ku, Osaka City, Osaka 545-8585, Japan, ^2^Molecular Probe Dynamics Laboratory, RIKEN Center for Molecular Imaging Science, 6-7-3 Minatojima-minamimachi, Chuo-ku, Kobe City, Hyogo 650-0047, Japan, ^3^Department of Medical Science on Fatigue, Osaka City University Graduate School of Medicine, 1-4-3 Asahimachi, Abeno-ku, Osaka City, Osaka 545-8585, Japan, ^4^Department of Clinical, Health and Special Needs Education Needs, Hyogo University of Teacher Education Graduate School of Education, 942-1 Shimokume, Kato City, Hyogo 673-1494, Japan.

## Competing interests

The authors declare that they have no competing interests.

## Authors' contributions

KM took part in the planning and designing of the experiment and cognitive tests, data analyses and manuscript preparation. MT contributed to the design and planning of the experiment and cognitive tests, data analyses and manuscript preparation. SF, EY and YS contributed to the design and planning of the experiment and in data preparation. KIM contributed to the design and planning of the experiment and recruited the participants. YW took part in the planning and design of the experiment and cognitive tests and helped write the manuscript. All authors read and approved the final manuscript.
